# ^68^Ga-pentixafor PET/CT Is a Supplementary Method for Primary Aldosteronism Subtyping Compared with Adrenal Vein Sampling

**DOI:** 10.1007/s11307-024-01976-0

**Published:** 2024-12-23

**Authors:** Tieci Yi, Difei Lu, Yonggang Cui, Zheng Zhang, Xing Yang, Jianhua Zhang, Lin Qiu, Haoyu Weng, Lin Liu, Xiaojiang Duan, Guangyu Zhao, Wei Ma, Ying Gao, Yan Fan

**Affiliations:** 1 Department of Cardiology, Peking University First Hospital, Beijing, China; 2 Institute of Cardiovascular Disease, Peking University First Hospital, Beijing, China; 3 Hypertension Precision Diagnosis and Treatment Research Center, Peking University First Hospital, Beijing, China; 4 Department of Endocrinology, Peking University First Hospital, Beijing, China; 5 Department of Nuclearuclear Medicine, Peking University First Hospital, Beijing, China; 6 Department of Urology, Peking University First Hospital, Beijing, China; 7 Echocardiography Core Lab, Institute of Cardiovascular Disease, Peking University First Hospital, Beijing, China

**Keywords:** CXCR4, ^68^Ga-pentixafor PET/CT, AVS, Primary aldosteronism, Subtyping

## Abstract

**Purpose:**

To investigate the diagnostic efficacy of ^68^Ga-pentixafor positron emission tomography/computed tomography (PET/CT) in primary aldosteronism (PA) subtyping and lateralization of aldosterone secretion in PA patients.

**Procedures:**

37 patients who were diagnosed with PA, were prospectively enrolled in the study, and underwent adrenal vein sampling (AVS) after ^68^Ga-pentixafor PET/CT was conducted. Lateralization index (LI), defined as aldosterone/cortisol ratio in the dominant side to the contralateral adrenal vein when bilateral adrenal vein catheterization succeeded, and the aldosterone/cortisol ratio in the left adrenal vein to IVC (LAV/IVC) when the catheterization of right adrenal vein failed, were applied to determine lateralization side. Statistical analysis was performed using SPSS 21.0.

**Results:**

The female proportion of all patients with PA was 32.4% (12/37), and the mean age was 51.3 ± 10.9 years. Patients with bilateral adrenal mass accounted for 54.1% (20/37), and 10 of them (27.0%) had adrenal hyperplasia or adrenal nodules ≤ 1.0 cm. In all 37 patients, the sensitivity, specificity and accuracy of ^68^Ga-pentixafor PET/CT in distinguishing lateralization by visualization were 89.3%, 77.8% and 86.5%, respectively. The area under the ROC curve for detecting positive lateralization based on the value of ^68^Ga-pentixafor SUV_max_ was 0.750 (95%CI 0.578–0.922, *p* = 0.026). The optimum SUV_max_ cut-off value was 6.86, with the sensitivity of 78.6%, specificity of 66.7%, and accuracy of 78.4%. Defining SUV ratio as SUV_max_/SUV of contralateral adrenal gland, the area under the ROC curve for identifying lateralization based on the SUV ratio was 0.710 (95%CI 0.500–0.921, *p* = 0.061). The optimum SUV ratio cut-off was 2.40, with the sensitivity of 60.7%, specificity of 88.9%, and accuracy of 67.6%. The consistency of ^68^Ga-pentixafor PET/CT with AVS was of no significant difference between patients with bilateral adrenal lesions (80.0%, 16/20) and unilateral lesion (94.1%, 16/17; *p* = 0.737), and no significance was revealed in the consistency between patients with adrenal hyperplasia or adrenal lesion of diameter ≤ 1 cm (81.8%, 9/11) and those with adrenal lesions > 1 cm (88.5%, 23/26; *p* = 0.884).

**Conclusions:**

^68^Ga-pentixafor PET/CT showed at least 80% consistency for the lateralization in patients with PA compared with AVS, even in those presented with bilateral adrenal hyperplasia. Visual analysis exhibited better diagnostic efficacy compared with SUV_max_ or SUV_max_/SUV of the contralateral adrenal gland.( ChiCTR2300073049. Registered 30 June 2023. Retrospectively registered)

**Supplementary Information:**

The online version contains supplementary material available at 10.1007/s11307-024-01976-0.

## Introduction

Primary aldosteronism (PA) is a major cause of secondary hypertension and accounts for approximately 6% of hypertensions [1]. Due to the heterogeneity of PA, subtyping classification, after case detection test and confirmatory tests, is of great importance because it determines the therapeutic plan [2]. For PA patients under 40-year-olds with unilateral adrenal adenoma, the diagnosis of aldosterone-producing adenoma (APA) is established and can be cured with adrenalectomy of the affected side [3]. Subtyping using computed tomography (CT) or magnetic resonance imaging (MRI) results in 37.8% misdiagnosis in lateralization determination [4].

Adrenal vein sampling (AVS), which is generally considered as the gold standard for PA subtyping, could be performed in PA patients with bilateral or unilateral masses to determine the lateralization, and the subtype of bilateral hyperaldosteronism (BHA) is an indication for long-term medical treatment [5]. Lateralization index (LI) is calculated as the side-to-side ratio of the aldosterone/cortisol ratio (ACR) of the adrenal veins of each side, thus it is mandatory for the accurate subtyping using AVS to the successful catheterization of adrenal veins (AVs) on both sides [6]. Since the success rate of catheterization varies from 30 to 96% [7], subtyping test other than AVS is in urgent need in clinical practice to localize potential APA.

CXC Chemokine receptor type 4 (CXCR4) is highly expressed in the zona glomerulosa of the adrenal cortex, a functional imaging technique of the CXCR4 ligand ^68^Ga-pentixafor PET/CT was subsequently developed for the diagnosis of APA [8]. Studies revealed that over 90% of CYP11B2-positive adrenal adenoma showed positive uptake on ^68^Ga-pentixafor PET/CT, mostly in APA with a diameter ≥ 1cm [9]. However, idiopathic hyperaldosteronism (IHA) accounts for 60% of PA population [10], and the diagnostic accuracy of ^68^Ga-pentixafor PET/CT in patients with PA, especially in IHA with adrenal masses of less than 1cm or adrenal hyperplasia in CT scan, is yet to be explored. Therefore, our study aimed to investigate the diagnostic efficacy of ^68^Ga-pentixafor PET/CT in PA subtyping and lateralization of aldosterone secretion in PA patients with bilateral and unilateral adrenal masses compared with AVS, and to explore a possible better diagnostic indicator of ^68^Ga-pentixafor PET/CT, including visual analysis or the maximum standardized uptake values (SUV_max_) for PA subtyping.

## Materials and Methods

### Design and Patients

**Fig. 1.**
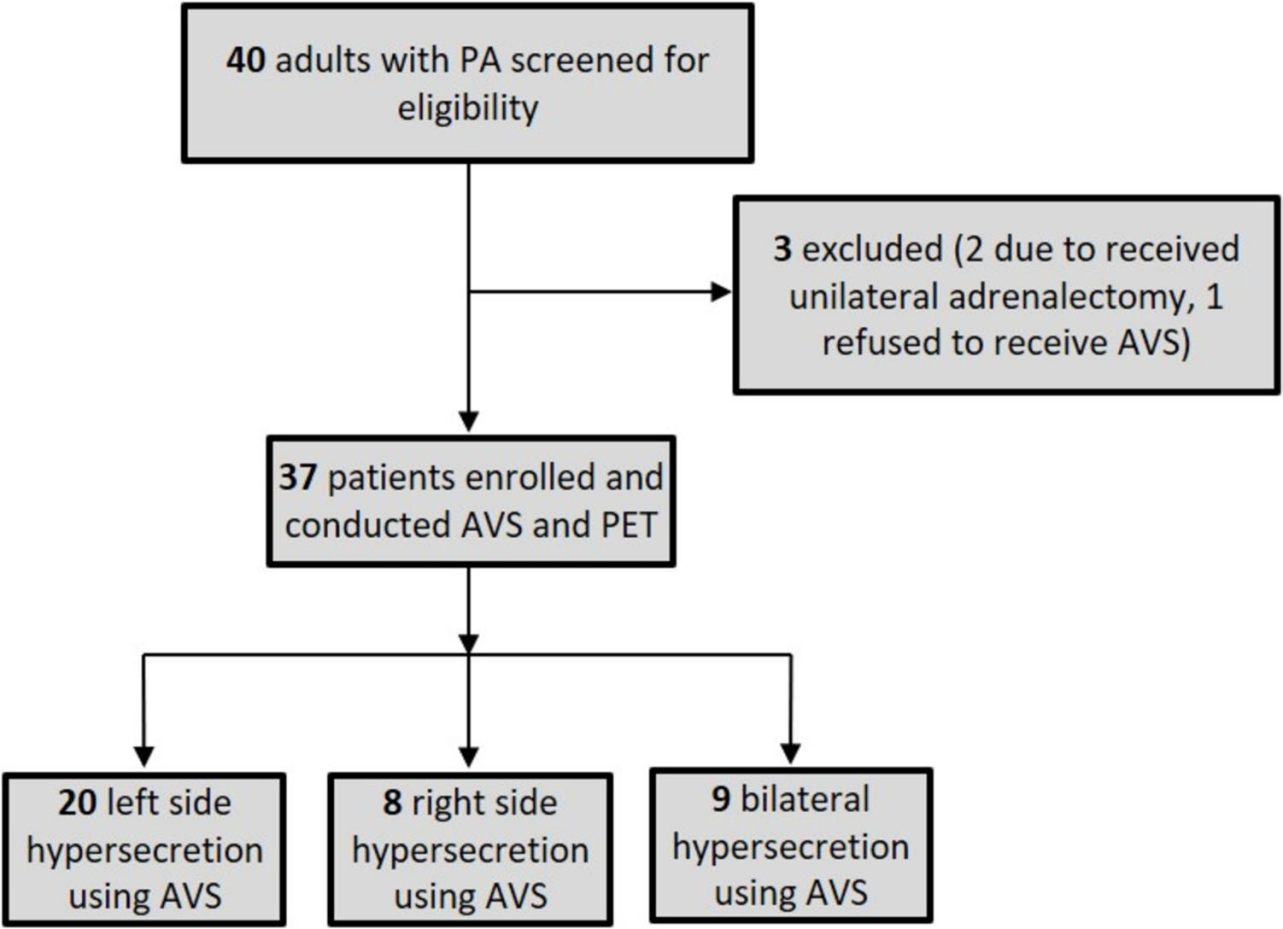
Flow chart of patient enrollment, results of AVS and ^68^Ga-Pentixafor PET/CT. PA, primary aldosteronism. PET, ^8^Ga-Pentixafor PET/CT. AVS, adrenal vein sampling

Patients with clinical suspected PA were prospectively enrolled in our study cohort at the Peking University First Hospital (PKUFH) (Fig. 1). The diagnostic criteria of PA were based on the clinical guidelines subcommittee of the Endocrine Society as followings [2]: (1) persistent hypertension with blood pressure > 160/100mmHg and/or hypokalemia; (2) aldosterone-to-renin ratio (ARR) > 3.7 (ng/dL)/(mU/L); (3) a positive captopril challenge test (CCT), which was considered when plasma aldosterone level decreased ≤ 30% after captopril administration, or a positive seated saline solution infusion test, which was achieved when plasma aldosterone concentration (PAC) > 10ng/dL after saline administration (500 ml/h for 4 h). A direct renin concentration (DRC) < 0.5mU/L was considered 0.5mU/L when calculating ARR. Individuals with severe renal or hepatic function impairment or during pregnancy or lactation were excluded. 1mg overnight dexamethasone suppression test was performed in all patients to rule out concurrent Cushing’s syndrome. All participants underwent abdominal CT scans to detect unilateral or bilateral adrenal nodules or hyperplasia. The study protocol was approved by the institutional review board of PKUFH (No.2021–461). All patients provided written informed consent prior to AVS and ^68^Ga-pentixafor PET/CT. Demographic data, duration of hypertension, the highest blood pressure, the lowest blood potassium level, standing DRC, PAC, ARR, confirmatory test, and adrenal imaging were collected.

### 68Ga-pentixafor PET/CT Scans

^68^Ga-pentixafor was produced by heating 3 mL 0.05 M HCl solution containing [^68^Ga]GaCl_3_ (632–1480 MBq), 200 μL of 1.0 M sodium acetate and 30 μg Pentixafor (10 μg/μL in DMSO) at 90 °C for 10 min.

After cooling down, the mixed solution was extracted with an activated C18 column (Waters Corporation, Milford, Massachusetts USA). The radiolabeled ligand was eluted by 0.5 mL 80% ethanol aqueous solution and was diluted with 5 mL saline. After purification, ^68^Ga-pentixafor (363–630 MBq) was obtained with > 99% radiochemical purity analyzed by radio-HPLC. In the injection, no impurities other than labeled and unlabeled peptides were seen.(Supplement Fig. 1.)

PET/CT images were collected by experienced physicians. Non-contrast enhanced CT scans were conducted over the adrenal region (120kV, 159mA, slice width 2mm), and PET images of the upper abdomen and adrenal region were obtained over 10 min after 25–30 min of an intravenous injection of ^68^Ga-pentixafor. The specific activity of the tracer is 12.1–21 MBq/μg, and the activity injected by the patient was calculated based on body weight, with 2.96 MBq of radiotracer injected per kilogram of body weight, which works out to be 0.14 to 0.24 µg of peptide mass injected per kilogram of body weight. A 192 × 192 matrix size and a 2.68 × 2.68 mm^2^ voxel size were used, and smoothed by a 4.5 mm full width at half-maximum Gaussian filter for all images. Two experienced board-certified nuclear medicine physicians (Cui Y and Fan Y), who remained blinded to AVS result of all patients, assessed the PEC/CT data. Lateralization using PET/CT was determined by comprehensive analysis including visual analysis, SUV_max_ of bilateral adrenal lesions, and SUV ratio, indicating SUV_max_ of dominant adrenal lesion/ SUV of contralateral adrenal gland.

### Subtyping Classification Using Adrenal Vein Sampling

Non-adrenocorticotropin (ACTH) stimulated—sequential AVS was conducted in all participants after ^68^Ga-pentixafor PET/CT scans. Successful catheterization is considered when cortisol levels in AVs to inferior vena cava (IVC) ratio (selective index, SI) > 2. Lateralization index (LI), which was defined as aldosterone/cortisol ratio in the dominant side to the contralateral AV, > 2 was considered as lateralization secretion of aldosterone or APA when CT scan indicated unilateral adrenal adenoma. The successful cannulation rate in the right adrenal vein is limited since the right adrenal vein drains directly into the IVC in an acute angle. Therefore, aldosterone/cortisol ratio in the left adrenal vein to IVC (LAV/IVC) was applied to determine lateralization side when the catheterization of right adrenal vein failed. LAV/IVC ≥ 5.5 was considered as left-sided hypersecretion, and < 0.5 as right-sided hypersecretion, with diagnostic sensitivity of 69% and specificity of 100% [11].

### Statistical Analysis

Continuous variables were expressed as mean ± standard deviation (SD) or median (IQR). Categorical variables were presented as percentage. The sensitivity, specificity and accuracy of ^68^Ga-pentixafor PET/CT results for detecting lateralization were calculated. The ROC curve of ^68^Ga-pentixafor PET/CT results was analyzed. Statistical analysis was performed using SPSS 21.0 (IBM Corp., NY, USA). *P* < 0.05 was considered of statistical significance.

## Results

### Baseline Characteristics

37 patients who were diagnosed with PA were enrolled in the study (Fig. 1), with female proportion of 32.4% (12/37) and mean age of 51.3 ± 10.9 years. The median duration of hypertension was 13.3 years, ranged from 0.1 to 53 years. The median of serum potassium was 2.92 mmol/L, ranged from 1.9 mmol/L to 4.03 mmol/L. Patients with bilateral adrenal mass accounted for 54.1% (20/37). The median of the maximum diameter of adrenal lesions was 1.50 cm, ranged from 0.8cm to 3.8cm. The median of the direct renin concentration (DRC) in standing position was 3.4 mU/L (0.5mU/L to 69.8mU/L, normal range 4.4–46.1 mU/L), and the median plasma aldosterone concentration (PAC) was 29.2 ng/dL (12.2ng/dL to 160.0ng/dL, normal range 3.0–35.3 ng/dL), with the aldosterone renin ratio (ARR) of 11.82 (normal range < 3.7). Captopril challenge test was performed in 35 patients, and the median of PAC after captopril challenge was 25.2 ng/dL, with the suppression ratio of PAC < 30% in all 35 patients. The saline solution infusion test, as a second confirmation test, was conducted in 12 patients, and median PAC after saline solution infusion was 16.2 ng/dL (> 10ng/dL was considered the confirmed diagnosis of PA). The main features of the population are shown in Table 1.

**Table 1:** Baseline characteristics and biochemical features of the 37 patients with PA

Variables	Value	Normal range
Age (year)	51.3 ± 10.9	-
Sex (male/female, n%)	25/12, 67.6%/32.4%	-
Maximum Systolic BP (mmHg)	173.1 ± 22.0	< 140
Maximum Diastolic BP (mmHg)	105.0 ± 18.7	< 90
Duration of hypertension (year)	13.3 ± 11.2	-
Serum potassium (mmol/L)	2.92 ± 0.48	3.5–5.5
Supine DRC (mU/L)	1.1 (0.5, 3.0)	2.8–39.9
Supine PAC (ng/dL)	29.0 (18.5, 41.2)	3.0–23.6
DRC in standing position (mU/L)	3.4 (1.3, 7.7)	4.4–46.1
PAC in standing position (ng/dL)	29.2 (22.3, 37.4)	3.0–35.3
ARR in standing position (ng/dL, mU/L)	11.8 (4.2, 22.1)	< 3.7
PAC after CCT (ng/dL, n = 35)	25.2 (14.2, 41.4)	-
PAC after saline solution infusion test (ng/dL, n = 12)	16.2 (13.2, 25.8)	< 5
Imaging of adrenal lesions (unilateral/bilateral, n%)	17/20, 45.9%/54.1%	-
Maximum diameter of adrenal mass (cm)	1.50 (1.05, 1.75)	-

Mean ± SD (age, BP, duration of hypertension, serum potassium), or median and IQ range. BP, blood pressure; PA, primary aldosteronism; DRC, direct renin concentration; PAC, plasma aldosterone concentration; ARR, aldosterone/renin ratio

### AVS Performance and Results

Sequential AVS was conducted in all patients, and the successful cannulation in the right adrenal vein was achieved in 26 patients (70.3%). Using LI in the 26 cases of successful bilateral catheterization and LAV/IVC in the 11 cases of the catheterization of right adrenal vein failed, 20 cases (54.1%) presented with left side hypersecretion, 8 of right side hypersecretion (21.6%), and 9 of bilateral hypersecretion (24.3%). Of all 37 cases experienced AVS, no adverse events including adrenal hemorrhage and contrast-induced nephropathy were reported.

### Effectiveness of ^68^Ga-Pentixafor PET/CT in Subtyping and Lateralization in PA Patients

**Fig. 2.**
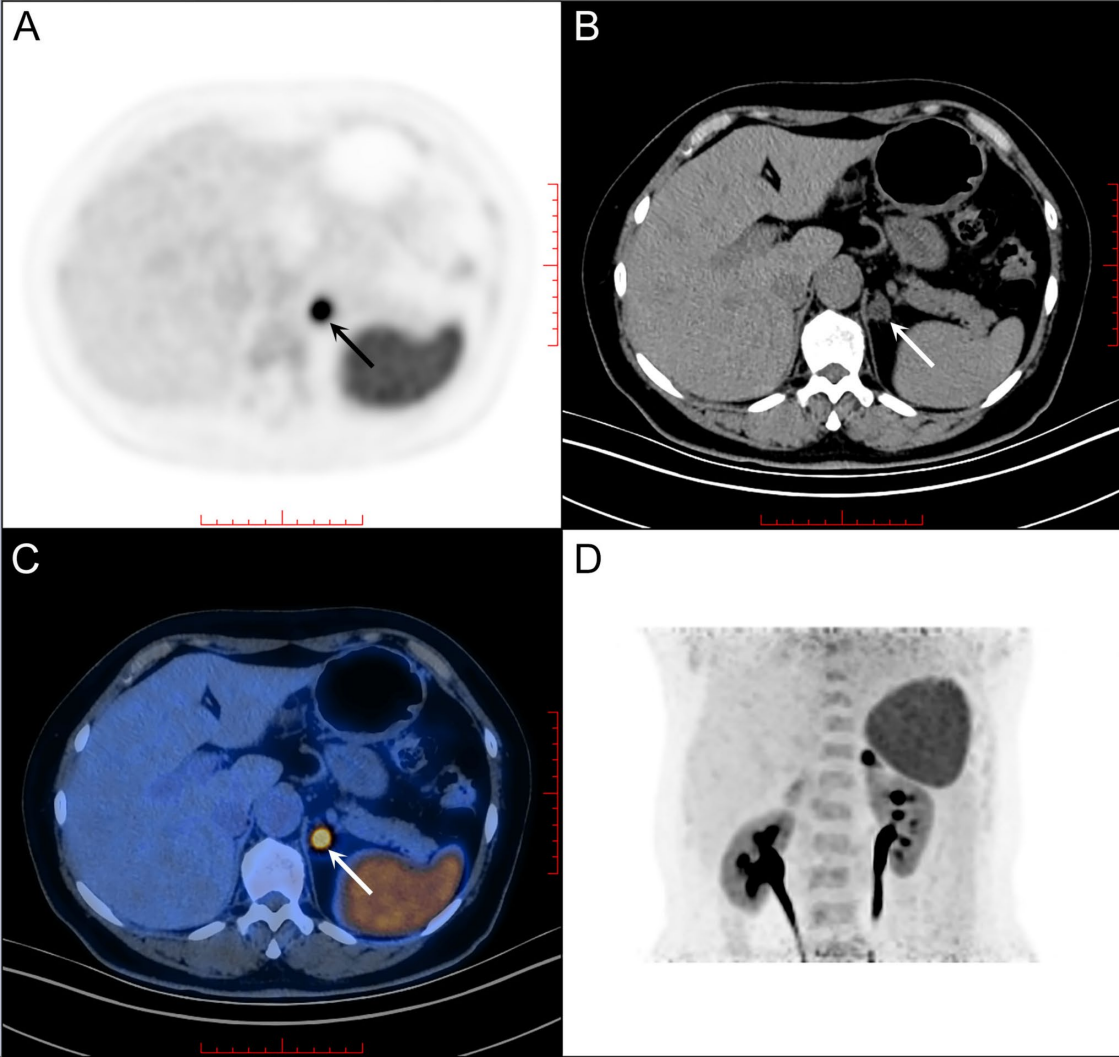
The performance of ^68^Ga-Pentixafor PET/CT imaging in PA patients. A 60-year-old woman presented with hypertension and hypokalemia of 2.45mmol/L. CT scan showed a nodule of 1.9 cm and the second nodule of 0.9 cm in the left adrenal gland, and another adrenal lesion of 1.0 cm on the right side. AVS indicated left aldosterone hypersecretion, with the lateralization index of 13.1 (> 2). **(B)** CT scan showed a nodule of 1.9 cm. **(A, C, D)** Positive findings of ^68^Ga-Pentixafor PET/CT demonstrated strong uptake of ^68^Ga-Pentixafor on the left adrenal lesion, with SUV_max_ of 13.9 in the nodule of 1.9 cm, which was in accordance with the findings of AVS. Left adrenalectomy was performed, and the plasma potassium increased to 4.64 mmol/L without oral supplement. PA, primary aldosteronism. AVS, adrenal vein sampling. (CT scan and 68Ga-Pentixafor PET/CT of the 0.9 cm nodule in the left adrenal gland and the 1.0 cm nodule on the right side are shown in Supplementary Material as Supplementary Fig. 2 & Fig. 3)

**Fig. 3.**
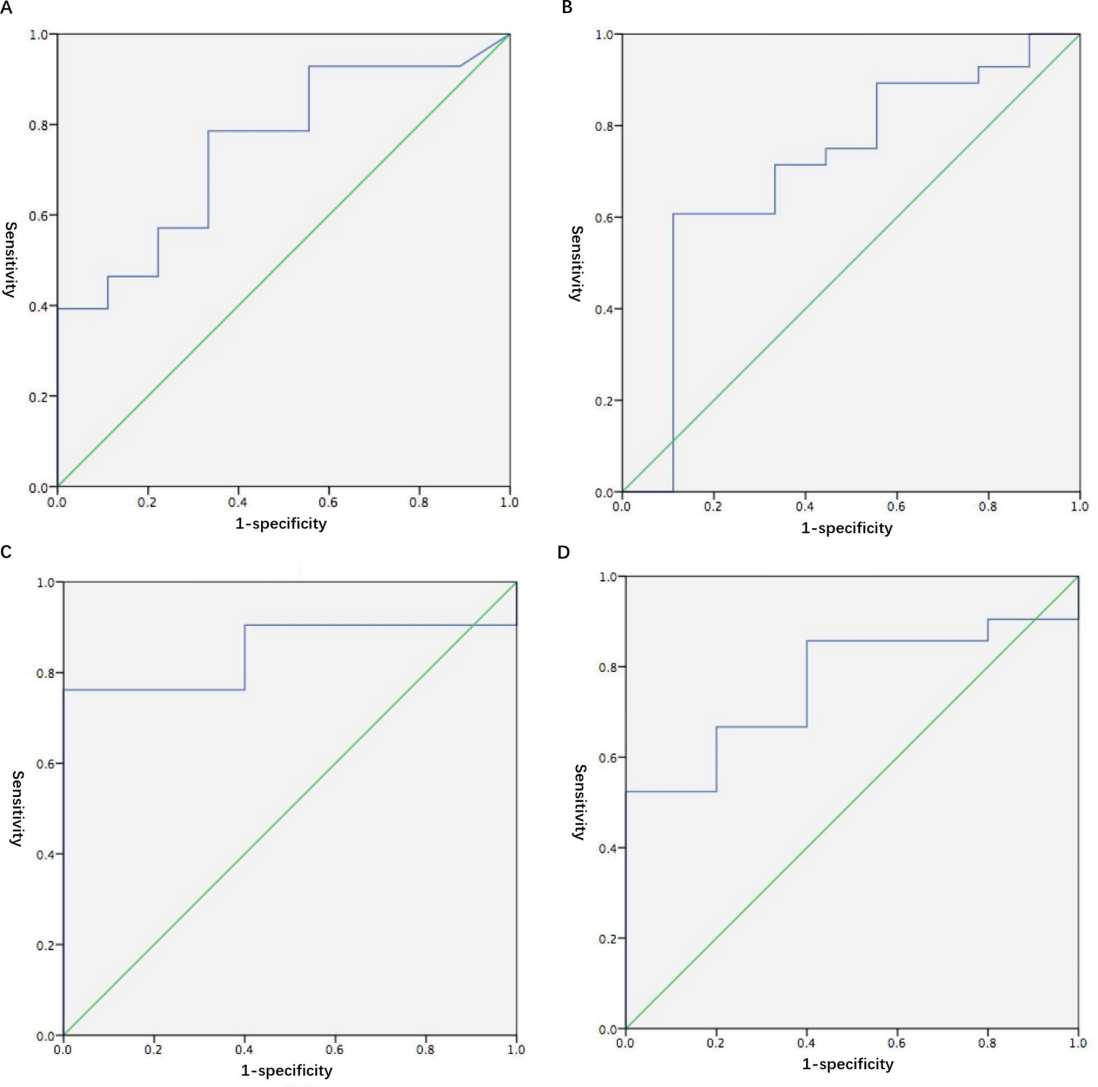
The ROC curve for lateralization using AVS. **(A)** The ROC curve for detecting positive lateralization based on the value of ^68^Ga-pentixafor SUV_max_ in 37 patients via LI or LAV/IVC. **(B)** The ROC curve for identifying lateralization according to the value of ^68^Ga-pentixafor SUV ratio in 37 patients via LI or LAV/IVC. **(C)** The ROC curve for detecting positive lateralization based on the value of ^68^Ga-pentixafor SUV_max_ in 26 patients via LI. **(D)** The ROC curve for identifying lateralization according to the value of ^68^Ga-pentixafor SUV ratio in 26 patients via LI. LI, lateralization index, defined as aldosterone/cortisol ratio in the dominant side to the contralateral adrenal vein. LAV/IVC, aldosterone/cortisol ratio in the left adrenal vein to intra vena cava

In 37 patients enrolled in this study, 54.1% (20/37) of them were presented with bilateral adrenal lesions (Fig. 2, Supplementary Figs. 2 & 3), and 11(29.7%) of them had adrenal hyperplasia or adrenal nodules ≤ 1.0 cm. The lateralization was confirmed via LI or LAV/IVC using AVS. The diagnostic accuracy based on visual analysis using ^68^Ga-pentixafor was 86.5%, and the sensitivity and specificity of ^68^Ga-pentixafor PET/CT in distinguishing lateralization by visualization were 89.3% and 77.8%, respectively. The area under the ROC curve (AUC) for detecting positive lateralization based on the value of ^68^Ga-pentixafor SUV_max_ was 0.75 (95%CI 0.57–0.92, *p* = 0.026, Fig. 3A). The optimum SUV_max_ cut-off value was 6.86, and the sensitivity was 78.6%, specificity was 66.7%, and accuracy was 78.4% at this cut-off value (Table 2). Defining SUV ratio as SUV_max_ of the higher side/SUV_max_ of contralateral adrenal gland, the AUC for identifying lateralization based on SUV ratio was 0.71 (95%CI 0.50–0.92, *p* = 0.061, Fig. 3B). The optimum SUV ratio cut-off was 2.40, and the sensitivity was 60.7%, specificity was 88.9%, and accuracy was 67.6% at this cut-off value (Table 2).

**Table 2:** Diagnostic efficiency of ^68^Ga-pentixafor PET/CT for positive lateralization based on visual and semi-quantitative analysis in all 37 patients with PA

Detective method	True positive	True negative	False positive	False negative	Sensitivity	Specificity	Accuracy
Visual analysis	25	7	2	3	89.3%	77.8%	86.5%
SUV_max_ = 6.86	23	6	3	5	78.6%	66.7%	78.4%
SUV_max_ = 5.86	26	5	5	1	92.9%	44.4%	83.8%
SUV ratio = 2.40	17	8	1	11	60.7%	88.9%	67.6%
SUV ratio = 1.56	19	7	3	8	71.4%	66.7%	70.3%

PA, primary aldosteronism

In the 26 patients with successful catheterization in the right adrenal vein, the lateralization was confirmed via LI. The sensitivity, specificity, and accuracy of ^68^Ga-pentixafor PET/CT in distinguishing lateralization by visualization were 85.7%, 100.0% and 88.5%, respectively (Table 3). The AUC for detecting positive lateralization based on the value of ^68^Ga-pentixafor SUV_max_ was 0.84 (95%CI 0.69–0.99, *p* = 0.018, Fig. 3C). The optimum SUV_max_ cut-off value was 6.86, the sensitivity was 76.2%, specificity was 100.0%, and the accuracy was 80.8% at this cut-off value (Table 3). The AUC for identifying lateralization based on the value of ^68^Ga-pentixafor SUV ratio was 0.76 (95%CI 0.56–0.95, *p* = 0.074, Fig. 3D). The optimum SUV ratio cut-off was 2.25, and the sensitivity was 52.4%, specificity was 100.0%, and accuracy was 61.5% at this cut-off value (Table 3).

**Table 3:** Diagnostic efficiency of ^68^Ga-pentixafor PET/CT for positive lateralization based on visual and semi-quantitative analysis in 26 patients with bilateral successful catheterization in AVS

Detective method	True positive	True negative	False positive	False negative	Sensitivity	Specificity	Accuracy
Visual analysis	18	5	0	3	85.7%	100.0%	88.5%
SUV_max_ = 6.86	16	5	0	5	76.2%	100.0%	80.8%
SUV_max_ = 5.86	19	3	2	2	90.5%	60.0%	84.6%
SUV ratio = 2.25	11	5	0	10	52.4%	100.0%	61.5%
SUV ratio = 1.56	14	4	1	7	66.7%	80.0%	69.2%

PA, primary aldosteronism; AVS, adrenal vein sampling

Using visualization of ^68^Ga-pentixafor PET/CT in distinguishing lateralization in 20 cases with bilateral adrenal lesions, the consistency of ^68^Ga-pentixafor PET/CT with AVS was 80.0% (16/20), which showed no significant difference compared with those with unilateral lesion (consistency, 94.1%, 16/17; *p* = 0.737). In the 11 cases with adrenal hyperplasia or adrenal lesion of diameter ≤ 1 cm, the consistency with AVS was 81.8% (9/11), and no significant difference was discovered compared with those with adrenal lesions over 1 cm (consistency, 88.5%, 23/26; *p* = 0.884).

## Discussion

Our study was designed as a prospective, single center, diagnostic study. Our findings revealed that ^68^Ga-pentixafor PET/CT achieved positive uptake and similar lateralization results in over 80% patient using AVS for subtyping, the gold standards for PA subtyping to date. Our conclusion suggested that visual identification of ^68^Ga-pentixafor PET/CT, with better diagnostic efficacy compared with SUV_max_ or bilateral SUV ratio, could be a supplementary tool for the subtyping of PA patients.

In previous studies, ^68^Ga-pentixafor PET/CT showed promising diagnostic efficacy in adrenal adenoma ≥ 1cm, and the SUV_max_ presented strong correlation with tumor size [12]. Previous studies enrolled patients with unilateral APA in comparison with nonfunctional adrenal adenoma (NFA) [9, 12], and the sensitivity of 88%—100%, specificity of 78.6%—100% and accuracy of 92.3% was achieved. However, the subtype of bilateral idiopathic adrenal hyperplasia (IAH) accounts for approximately 60% of all PA patients [13]. Therefore, whether ^68^Ga-pentixafor PET/CT could be conducted in individuals with PA and bilateral adrenal masses for seeking possible lateralization and the opportunity for a cure of hypertension and hypokalemia, was yet to be investigated.

AVS is considered as the gold standard method of subtyping in PA diagnosis [14]. However, the prevalence in clinical practice of AVS is limited due to the invasive process, high expense, and varied success rate in different sites. Non-invasive algorithm including biochemical data and demographic characteristics [15], biochemical tests [16], ACTH stimulating test [17], and functional imaging was developed to provide more clinical evidence for PA subtyping instead of AVS [18]. A prospective study enrolled 66 patients with APA, 33 with IHA and 21 with NFA, and AVS was performed in 11 patients [19]. ^68^Ga-pentixafor PET/CT exhibited a sensitivity of 95.0% in PA patients with unilateral lesions and a sensitivity of 92.3% in patients with bilateral adrenal lesions, and the consistency in subtyping comparing AVS and ^68^Ga-pentixafor PET/CT was 60% (3/5) in 5 cases with successful bilateral sampling [19]. A recent study prospectively compared AVS and ^68^Ga-pentixafor PET/CT in 26 patients with PA, whose long diameter of adrenal adenoma was 1.2cm in average, and the consistency of the two methods in subtyping was 77% (20/26), of whom 13 cases were unilateral primary aldosteronism (UPA) and 7 cases were bilateral primary aldosteronism (BPA) [20]. The above study did not provide the characteristics of CT scan and the information of bilateral or unilateral adrenal mass in the relatively small sample size. Therefore, our study confirmed the approximately 80% consistency of ^68^Ga-pentixafor PET/CT with AVS in subtyping in an expended consecutive prospective cohort of PA. In addition, the diagnostic accuracy was of no significant difference between patients with unilateral and bilateral adrenal lesions, or between cases of adrenal lesion ≤ 1 cm and > 1 cm, indicted that ^68^Ga-pentixafor PET/CT could be a non-invasive, supplementary with AVS, or even independent diagnosis method for subtyping in PA patients with adrenal hyperplasia or small adrenal lesions less than 1 cm.

Our study further explored the diagnostic value of visual analysis, SUV_max_, and bilateral SUV ratio in subtyping. Previous studies suggested that SUV_max_ of 7.3 to 11.18 achieved a specificity of 100% in APA patients with nodules greater than 1 cm in diameter [9, 12, 19]. Our study indicated that the SUV_max_ cut-off value of 6.86 exhibited a sensitivity of 78.6%, specificity of 66.7%, and accuracy of 78.4% for subtyping, with less diagnostic value compared with visual analysis. Further development of novel indicators of ^68^Ga-pentixafor PET/CT is in need for the accurate lateralization.

There are several limitations to this study. First, it was a single-center study, and the sample size was relatively small. A larger study population and multicenter study could be conducted to further confirm the results. Second, the subtyping results of the two methods were not confirmed using immunohistochemical detection of aldosterone synthase (CYP11B2) since only a small proportion of the enrolled patient underwent adrenalectomy, which may lead to bias to the results.

## Conclusions

Our findings revealed that ^68^Ga-pentixafor PET/CT displayed an at least 80% consistency for the lateralization in patients with PA compared with AVS, even in those presented with bilateral adrenal hyperplasia. Visual analysis suggested better diagnostic efficacy compared with SUV_max_ or SUV_max_/SUV of the contralateral adrenal gland.

## Supplementary Information

Below is the link to the electronic supplementary material.

Supplementary file1 (DOCX 1903 KB)

## Data Availability

The main data presented in this study are available in the article. Detailed information are available on reasonable request from the corresponding author.
