# Supplementary material for: 68Ga-pentixafor PET/CT Is a Supplementary Method for Primary Aldosteronism Subtyping Compared with Adrenal Vein Sampling
Source: Mol Imaging Biol. 2024 Dec 23;27(1):142–50. doi: 10.1007/s11307-024-01976-0 (PMC11805762; doi:10.1007/s11307-024-01976-0)
Supplement: Supplementary file 1 — Supplementary file1 (DOCX 1903 KB) [file 11307_2024_1976_MOESM1_ESM.docx]

**Supplementary Material**


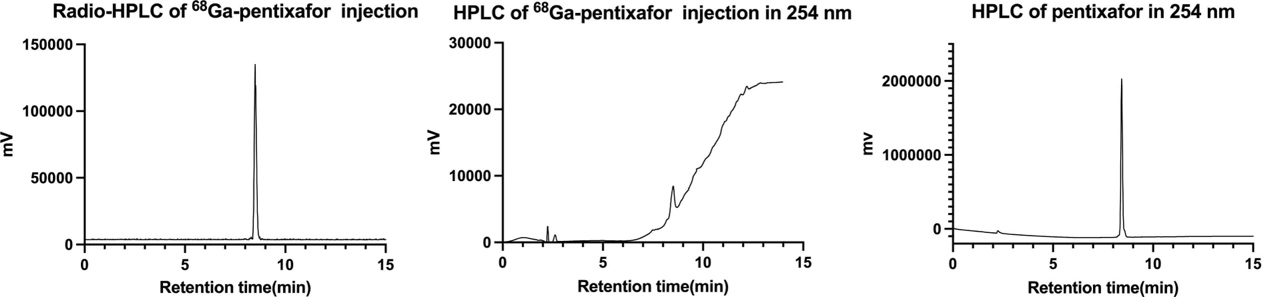


**Supplementary Figure 1.** HPLC chromatograms of the radiotracer. The radiochemical purity of the ^68^Ga-pentixafor is greater than 99%. In the injection, no impurities other than labeled and unlabeled peptides were seen.


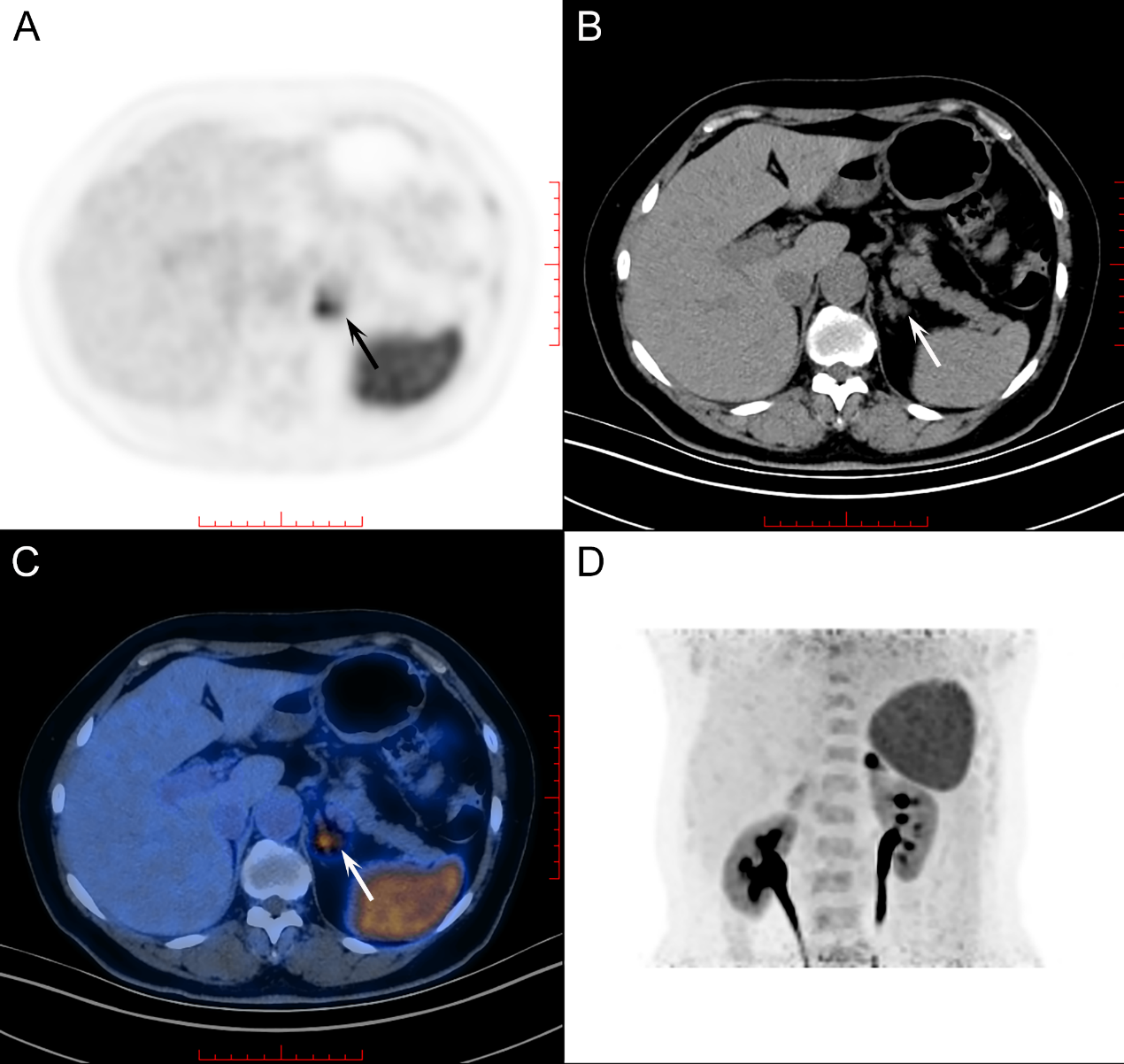


**Supplementary Figure 2.** The performance of ^68^Ga-Pentixafor PET/CT imaging in PA patients. A 60-year-old woman presented with hypertension and hypokalemia of 2.45mmol/L. Except for the 1.9cm nodule showed in Fig. 2 of the main text, CT scan also showed a second nodule of 0.9 cm in the left adrenal gland, and another adrenal lesion of 1.0 cm on the right side. **(B)** CT scan showed a nodule of 0.9 cm. **(A, C, D)** ^68^Ga-Pentixafor PET/CT showed no strong uptake of ^68^Ga-Pentixafor on this left adrenal lesion. PA, primary aldosteronism. AVS, adrenal vein sampling.


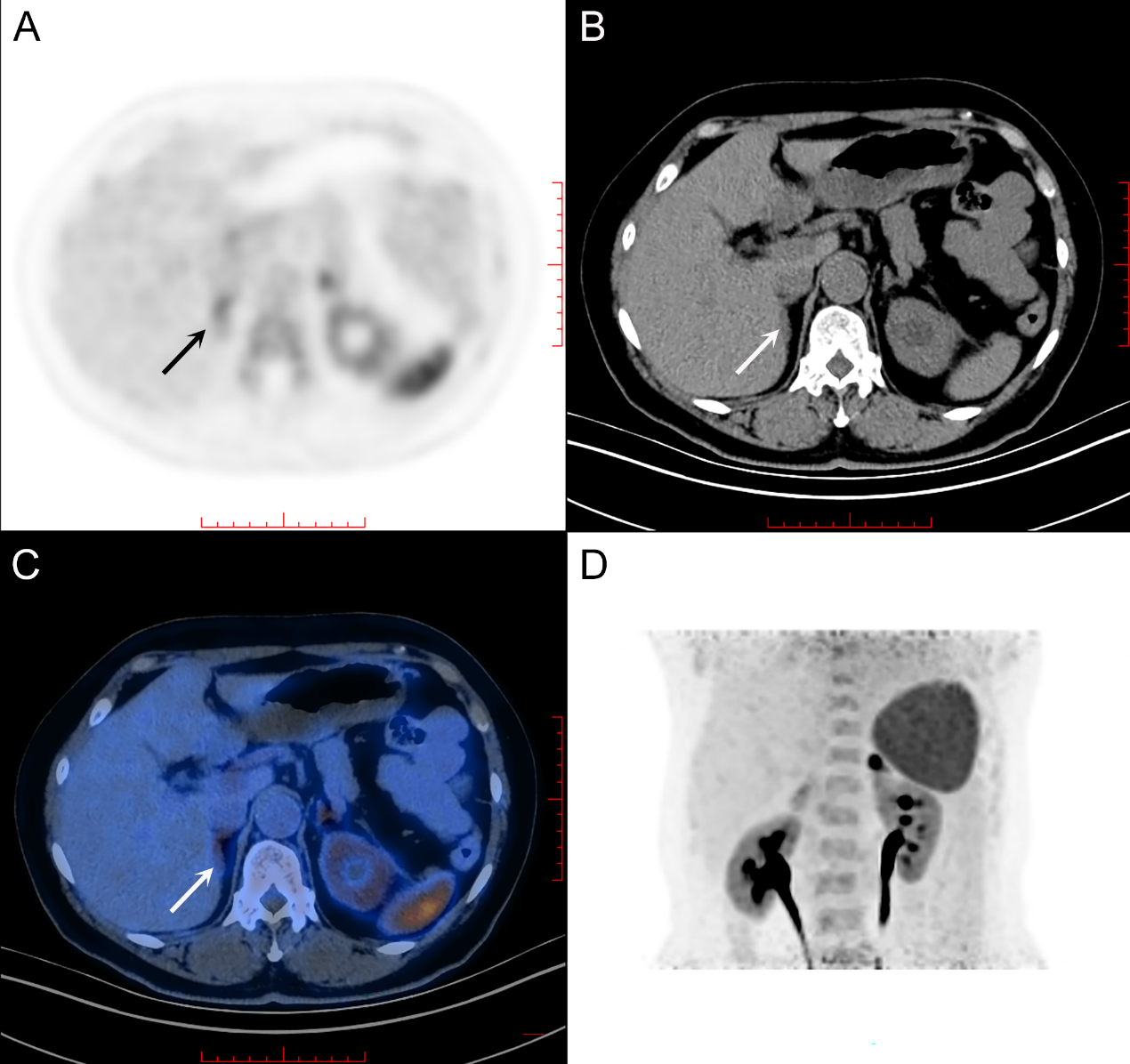


**Supplementary Figure 3**. The performance of ^68^Ga-Pentixafor PET/CT imaging in PA patients. A 60-year-old woman presented with hypertension and hypokalemia of 2.45mmol/L. Except for the 1.9cm nodule showed in Fig. 2 of the main text, CT scan also showed a second nodule of 0.9 cm in the left adrenal gland, and another adrenal lesion of 1.0 cm on the right side. **(B)** CT scan showed a lesion on the right side. **(A, C, D)** ^68^Ga-Pentixafor PET/CT showed no strong uptake of ^68^Ga-Pentixafor on this right adrenal lesion. PA, primary aldosteronism. AVS, adrenal vein sampling.


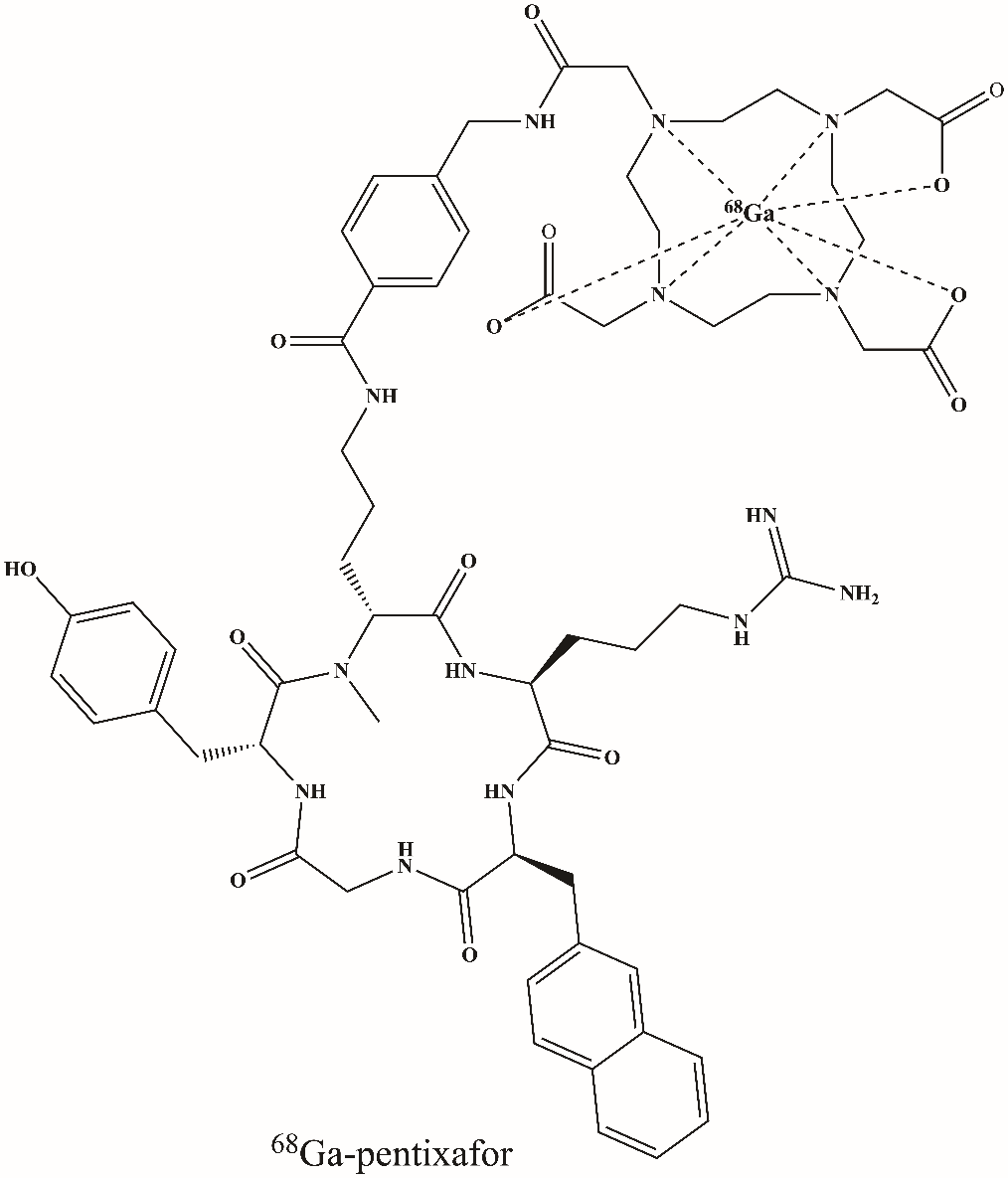


**Supplementary Figure 4.** The chemical structure diagram for ^68^Ga-pentixafor.
